# Supplementary material for: Growth Response and Differentiation of Bone Marrow-Derived Mesenchymal Stem/Stromal Cells in the Presence of Novel Multiple Myeloma Drug Melflufen
Source: Cells. 2022 May 7;11(9):1574. doi: 10.3390/cells11091574 (PMC9103864; doi:10.3390/cells11091574)
Supplement: Supplementary file 1 [file cells-11-01574-s001.zip › Supplementary material 00.pdf]

Supplementary data

Table S1: BMSC donor information marker expression.

Donor ID	1	2	3	4	5	6	7	8
**Sex**	Male	Female	Female	Male	Male	-	-	Female
**Age**	90	87	91	92	82	-	-	56
**BMI**	24.2	28.9	23.4	19.0	25.6	-	-	48.4

- donor information unknown

Table S2: BMSC surface marker expression.

Donor	1	2	3	4	5	6	7	8
**Passage**	2	2	3	3	3	3	2	3
**CD14**	1.2 %	2.1 %	7.4 %	4.1 %	5.2 %	2.6 %	3.4 %	1.7 %
**CD19**	1.2 %	2.4 %	7.1 %	2.5 %	2.0 %	1.1 %	5.3 %	1.6 %
**CD34**	0.8 %	0.5 %	3.1 %	2.1 %	1.0 %	0.4 %	1.4 %	1.5 %
**CD45**	1.6 %	1.0 %	8.8 %	6.2 %	3.5 %	0.9 %	2.7 %	2.4 %
**CD73**	100.0 %	100.0 %	95.4 %	95.6 %	95.6 %	100.0 %	100.0 %	100.0 %
**CD90**	98.0 %	89.4 %	90.2 %	94.2 %	86.6 %	88.0 %	97.6 %	99.8 %
**CD105**	100.0 %	99.9 %	93.6 %	98.7 %	97.2 %	100.0 %	100.0 %	99.9 %
**HLA-DR**	97.1 %	88.7 %	92.2 %	88.6 %	96.8 %	95.7 %	72.0 %	96.0 %

Table S3: Antibodies used in immunocytochemical staining.

Osteogenically differentiated BMSCs

Type	Antibody	Host species	Clone	Dilution
Primary	Anti-Collagen I (ab260043)[1]	Rabbit	EPR22894-89	1:250
Primary	Anti-Osteocalcin (MAB1419)[2]	Mouse	IgG1 Clone #190125	1 : 50
Secondary	Anti-rabbit IgG Alexa fluor 488 (A21206)[3]	Donkey		1 : 500
Secondary	Anti-mouse IgG1 Alexa fluor 488 (A21121)[3]	Goat		1 : 500

Angiogenesis assay

Type	Antibody	Host species	Clone	Dilution
Primary	Anti- α-smooth muscle actin (ab7817)[1]	Mouse	1A4	
Secondary	Anti-mouse IgG (H+L) Alexa Fluor 568 (A11031)[3]	Goat		1:500

1 Abcam, Cambridge, United Kingdom. 2R&D systems, Minneapolis, MN, US. 4 Sigma-Aldrich., 3 Thermo Fisher Scientific

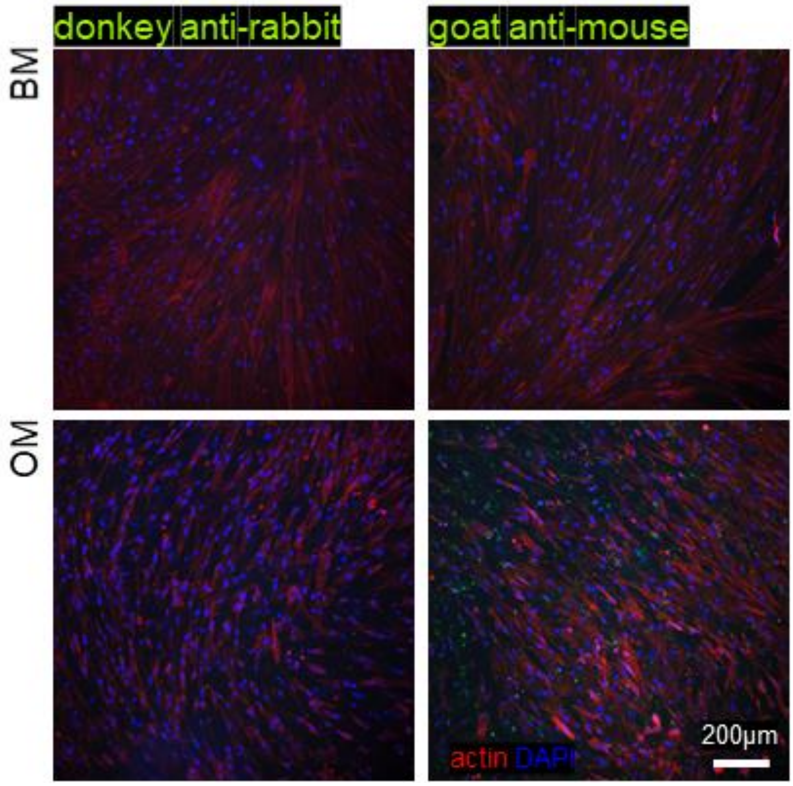

Figure S1: Secondary antibody control images showing an unsignificant amount of background staining by the secondary antibodies in absence of any primary antibody. BM = basic medium, OM = osteogenic medium. Scale bar: 200µm.

Table S4: Primer sequences for qRT-PCR.

**Gene**	**Accession number**	**5′-Sequence-3′**	**Product size (bp)**
DLX5	NM_005221.5	Forward ACCATCCGTCTCAGGAATCG<br>Reverse CCCCCGTAGGGCTGTAGTAGT	75
FABP4	NM_001442	Forward GGTGGTGGAATGCGTCATG<br>Reverse CAACGTCCCTTGGCTTATGC	71
LEP	NM_000230	Forward ACAATTGTCACCAGGATCAATGAC<br>Reverse TCCAAACCGGTGACTTTCTG	73
RPLP0	NM_001002	Forward AATCTCCAGGGGCACCATT<br>Reverse CGCTGGCTCCCACTTTGT	70
RUNX2A	NM_001024630.3	Forward CTTCATTCGCCTCACAAACAAC<br>Reverse TCCTCCTGGAGAAAGTTTGCA	62
SP7	AF477981	Forward TGAGCTGGAGCGTCATGTG<br>Reverse TCGGGTAAAGCGCTTGGA	79

bp: base pair

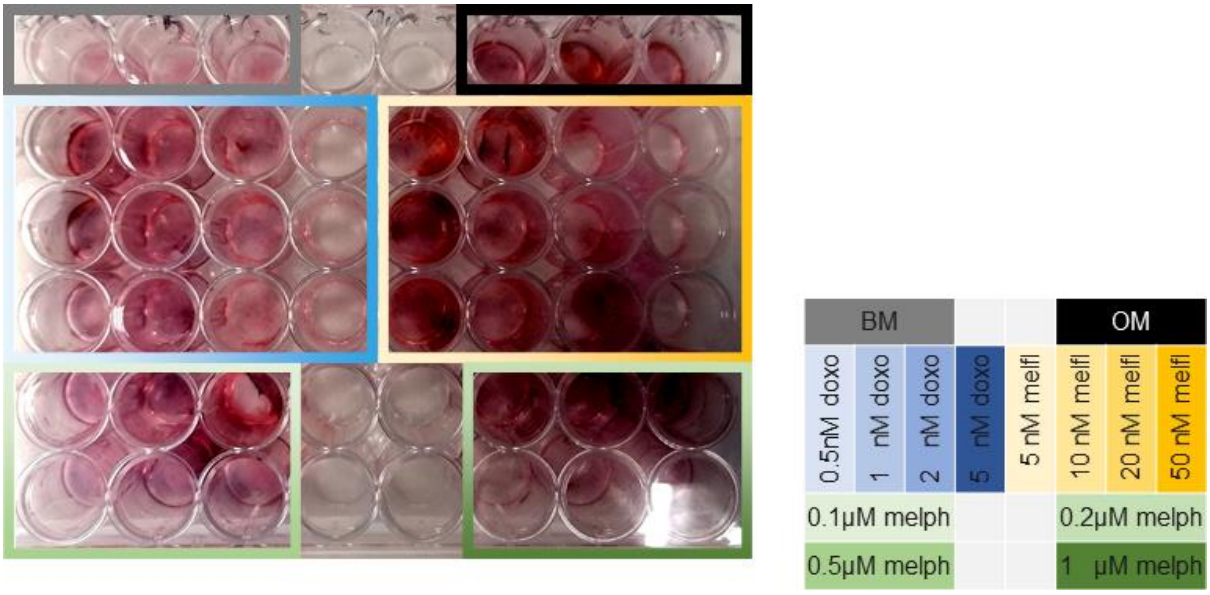

Figure S2: Representative image of Alizarin Red S stainings for the mineralized matrix from a culture well plate with osteogenically differentiated BMSC at 21 days of culture with drugs added at the indicated concentrations. melfl = melflufen, melph = melphalan, doxo = doxorubicin

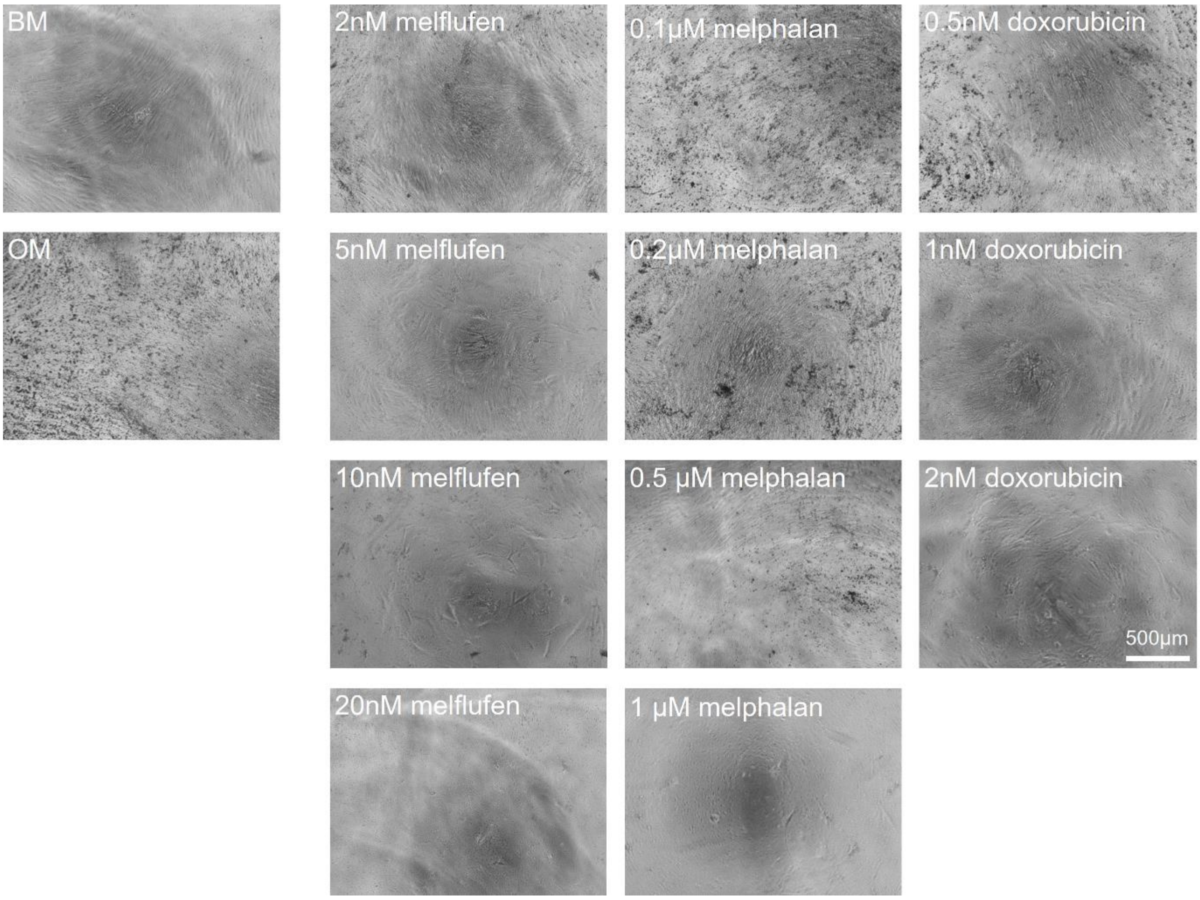

Figure S3: Phase contrast images of osteogenically differentiated BMSCs at 21 days of culture. BM = basic medium control, OM = osteogenic medium control without drugs. Scale bar: 500µm
